# Granulomatous pigmented purpuric dermatosis: report of a Latin-American case with blaschkoid distribution^☆^^☆☆^

**DOI:** 10.1016/j.abd.2019.09.002

**Published:** 2019-09-30

**Authors:** Daniela Carvajal, Claudia Quiroz, Claudia Morales, Javier Fernández

**Affiliations:** a Dermatology Department, Faculty of Medicine, University of Chile, Santiago, Chile; b Dermatology Unit, Clinical Hospital University of Chile, Santiago, Chile; c Pathology Unit, Clinical Hospital University of Chile, Santiago, Chile; d Dermatology Unit, Hospital San José, Santiago, Chile

**Keywords:** Dyslipidemias, Granuloma, Skin diseases, vascular

## Abstract

Granulomatous pigmented purpuric dermatosis clinically manifests as hyperpigmented maculae and petechiae, predominantly on the lower extremities. Histopathologically, it is characterized by a lymphocytic infiltrate in the upper dermis, extravasated erythrocytes, and hemosiderin deposits. There is an infrequent variant called granulomatous pigmented purpuric dermatosis, which histologically is characterized by the presence of non-necrotizing granulomas associated with the classic findings of other pigmented purpuric dermatoses. It more frequently affects middle-aged women of Asian origin, and predominantly on the lower extremities. The authors present the case of a female patient with granulomatous pigmented purpuric dermatosis on the lower extremities with blaschkoid distribution.

## Introduction

Pigmented purpuric dermatosis (PPD) or capillaritis represents a heterogeneous group of dermatoses of uncertain etiology, which are characterized by pigmented red to brown maculae and petechiae, predominantly on the lower extremities. Classically, five clinical variants of PPD have been described: purpura annularis telangiectodes of Majocchi, progressive pigmentary dermatosis of Schamberg, pigmented purpuric dermatitis of Gougerot and Blum, eczematoid-like purpura of Doucas and Kapentanakes, and lichen aureus.^1^ The classic histological findings include a superficial perivascular lymphocytic infiltrate, erythrocyte extravasation, and hemosiderin deposit, without vasculitis. A granulomatous variant of PPD has been recently described, whose main histological characteristic is the presence of non-necrotizing granulomas superimposed with the classic findings of PPD.^2^

This report details the case of a female Latin-American patient with hyperpigmented patches on the lower extremities, some of them with a blaschkoid distribution, whose skin biopsy revealed the presence of non-necrotizing granulomas associated with the common features of PPD.

## Case report

A 62-year-old woman presented with a six-month history of asymptomatic pigmented patches on both legs. She also had a history of diabetes mellitus II, hypertension, dyslipidemia, and hypothyroidism, under treatment with metformin, losartan, atenolol, atorvastatin, and levothyroxine. There were no other associated symptoms or changes in the usual medication.

**Figure 1:**
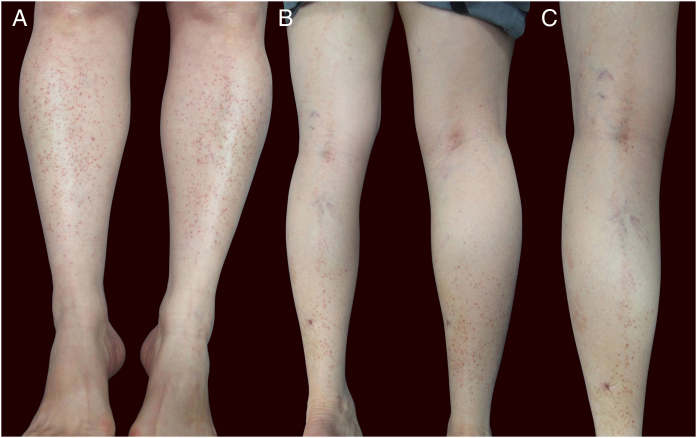
(A) Patient with red-brown patches and petechiae symmetrically distributed on the lower extremities. (B and C) Lesions on the posterior aspect of the left leg in a linear disposition, with blaschkoid distribution.

**Figure 2:**
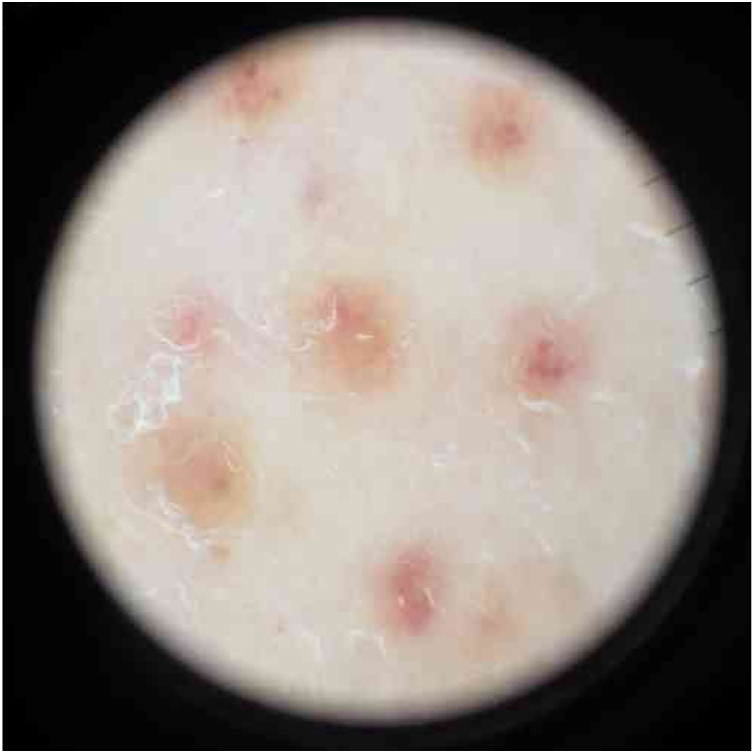
Dermatoscopy of the lesions showed red-brown spots and globules arranged on a background of coppery-red pigmentation.

Physical examination showed small red-brown patches of 2–3 mm and non-palpable petechiae symmetrically distributed on the lower extremities (Fig. 1A). In the posterior aspect of the left leg, the lesions had a linear disposition with a blaschkoid distribution (Fig. 1B and C). Dermatoscopy showed red-brown spots and globules arranged on a background of coppery-red pigmentation (Fig. 2).

**Figure 3:**
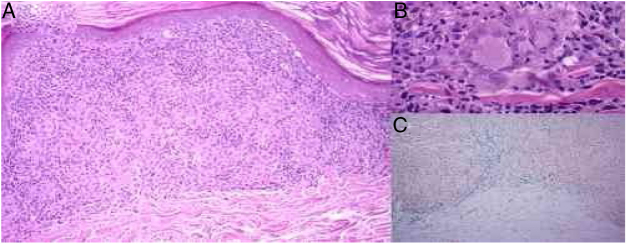
(A) Thinned epidermis, lichenoid infiltrate with lymphocytes and histiocytes forming non-necrotizing granulomas in the dermis and erythrocyte extravasation (hematoxylin & eosin, ×100). (B) Small non-necrotizing granulomas in the dermis formed by deposits of epithelioid cells and multinucleated giant cells (hematoxylin & eosin, ×400). (C) Erythrocyte extravasation and hemosiderin deposits in the dermis, especially under the inflammatory deposits with granulomas (Prussian blue iron stain, ×100).

Skin biopsy showed a thinned epidermis, lichenoid infiltrate in bands with lymphocytes in the papillary and reticular dermis (Fig. 3A), small non-necrotizing granulomas in the dermis formed by deposits of epithelioid cells and multinucleated giant cells, surrounded by lymphoplasmacytic cells, without necrosis (Fig. 3B). Erythrocyte extravasation and a few eosinophils were observed, with the absence of vasculitis. Hemosiderin deposits were demonstrated using Prussian blue iron stain (Fig. 3C). Periodic acid–Schiff (PAS) and Ziehl–Neelsen staining were negative.

Laboratory studies showed elevated low-density lipoprotein cholesterol and triglycerides. C-reactive protein and erythrocyte sedimentation rate were normal, without organ dysfunction. The clinical history and histological findings confirmed the diagnosis of granulomatous PPD.

## Discussion

Granulomatous PPD was first described by Saito and Matsuoka in 1996, representing the last variety of PPD published.^2^ To date, only 27 cases of granulomatous PPD have been published, with a clear predominance in females (74%), between 9 and 75 years of age, and with half of the cases in Asian patients.^3^ In recent years, reports of granulomatous PPD have increased in Caucasian patients, with no cases published among the Latin-American population.

Clinically, granulomatous PPD presents as red-brown patches and petechiae, similar to other forms of PPD, with the lower extremities being the most frequent location.^4^ The dermatoscopic findings in the present case are similar to other reports, describing multiple round-to-oval brown-red dots, globules, and patches in a background of non-networked coppery-red pigmentation.^5^ There have been reports of linear unilateral capillaritis, lichen aureus, and Schamberg disease with lesions arranged in a linear distribution.^2^, ^6^ However, there are no reports to date of granulomatous PPD with a linear or blaschkoid distribution.

Histologically, granulomatous PPD presents the classic findings of other forms of PPD, with the addition of the presence of lymphohistiocytic infiltrate and the formation of non-necrotizing granulomas in the papillary dermis, some arranged around the vascular plexus.^2^, ^3^ Other findings include vacuolar changes or lichenoid infiltrate in the dermoepidermal junction.^4^, ^7^ The histopathologic differential diagnosis includes other diseases that present granulomas; infectious diseases such as tuberculosis or atypical mycobacteria, and non-infectious disease such as sarcoidosis, drug reaction, metastatic Crohn's disease, granulomatous vasculitis, or mycosis fungoides.^5^

The etiology of granulomatous PPD is still uncertain. This variant has been associated with hyperlipidemia in more than 50% of the cases.^3^ It has been postulated that hyperlipidemia could cause chronic inflammation and an insufficient Th1 response, thus leading to granuloma formation.^5^ Other theories postulate that underlying vascular injury, induced by lipid deposition in endothelial cells, might result in a granulomatous response.^4^

Autoimmunity is a common finding in patients with granulomatous PPD, being reported in more than half of the cases. The most frequent are hypothyroidism, ulcerative colitis, Sjögren's syndrome, and multiple sclerosis.^3^, ^8^ Some cases present positive self-immunity markers such as antinuclear antibodies, rheumatoid factor, and cryoglobulins, suggesting that autoimmunity might also play a role in this variant. Further studies are necessary to establish an association.^3^ Other frequent findings described are arterial hypertension and diabetes mellitus.^2^

Granulomatous PPD is an often asymptomatic, benign condition.^2^ Thus, treatment is usually reserved for the management of associated symptoms, such as pruritus, and for cosmetic reasons, in patients distressed by the appearance of their skin. Management of suspected triggers such as dyslipidemia and the treatment of associated venous stasis, when present, might be beneficial.^2^, ^3^ Antihistamines are used to control pruritus; however, they do not have an effect on the course of the disease. A systematic review^9^ of the therapeutic strategies for PPD demonstrated that the use of local steroids, local calcineurin-inhibitors, rutoside, high doses of ascorbic acid, colchicine, pentoxifylline, phototherapy, and laser therapy yielded a good response. Cyclosporine A and others immunosuppressants can be used in refractory disease. Larger systematic studies are necessary to assess the effectiveness of the therapeutic strategies.

## Financial support

None declared.

## Author's contribution

Daniela Carvajal, Claudia Quiroz, Claudia Morales and Javier Fernández: Approval of the final version of the manuscript; conception and planning of the study; elaboration and writing of the manuscript; obtaining, analyzing and interpreting the data; effective participation in research orientation; intellectual participation in propaedeutic and/or therapeutic conduct of the cases studied; critical review of the literature; critical review of the manuscript.

## Conflicts of interest

The authors declare no conflicts of interest.

## References

[1] Ramos-Rodríguez C., García-Arpa M., Gónzalez-López L., Relea M.F. (2015). Granulomatous pigmented purpuric dermatosis: a temporary case?. Rev Esp Patol.

[2] Allan A., Altman D.A., Su W. (2017). Granulomatous pigmented purpuric dermatosis. Cutis.

[3] García-Rodiño S., Rodríguez-Granados M.T., Seoane-Pose M.J., Espasandín-Arias M., Barbeito-Castiñeiras G., Suárez-Peñaranda J.M. (2017). Granulomatous variant of pigmented purpuric dermatosis: report of two cases and review of the literature. J Dtsch Dermatol Ges.

[4] Morrissey K., Rosenbach M., DeHoratius D., Elenitsas R., Tetzlaff M.T. (2014). Granulomatous changes associated with pigmented purpuric dermatosis. Cutis.

[5] MacKenzie A.I., Biswas A. (2015). Granulomatous pigmented purpuric dermatosis: report of a case with atypical clinical presentation including dermoscopic findings. Am J Dermatopathol.

[6] Ma H.J., Zhao G., Liu W., Dang Y.P., Li D.G. (2007). Unilateral linear capillaritis: two unusual Chinese cases. Eur J Dermatol.

[7] Battle L.R., Shalin S.C., Gao L. (2015). Granulomatous pigmented purpuric dermatosis. Clin Exp Dermatol.

[8] Wakasuwa C., Fujimura T., Haga T., Aiba S. (2013). Granulomatous pigmented purpuric dermatitis associated with primary Sjögren's syndrome. Acta Derm Venereol.

[9] Plachouri K.M., Florou V., Georgiou S. (2018). Therapeutic strategies for pigmented purpuric dermatoses: a systematic literature review. J Dermatol Treat.

